# Cancer Progression Mediated by Horizontal Gene Transfer in an *In Vivo* Model

**DOI:** 10.1371/journal.pone.0052754

**Published:** 2012-12-28

**Authors:** Catalina Trejo-Becerril, Enrique Pérez-Cárdenas, Lucía Taja-Chayeb, Philippe Anker, Roberto Herrera-Goepfert, Luis A. Medina-Velázquez, Alfredo Hidalgo-Miranda, Delia Pérez-Montiel, Alma Chávez-Blanco, Judith Cruz-Velázquez, José Díaz-Chávez, Miguel Gaxiola, Alfonso Dueñas-González

**Affiliations:** 1 Division of Basic Research, Instituto Nacional de Cancerología, Mexico City, Mexico; 2 OncoXL, Geneva, Switzerland; 3 Department of Pathology, Instituto Nacional de Cancerología, Mexico City, Mexico; 4 Instituto de Física, Universidad Nacional Autónoma de México/Instituto Nacional de Cancerología, Mexico City, Mexico; 5 Cancer Genomics Laboratory, Instituto Nacional de Medicina Genómica, Mexico City, Mexico; 6 Department of Cytogenetics, Instituto Nacional de Cancerología, Mexico City, Mexico; 7 Unidad de Investigación, Instituto Nacional de Enfermedades Respiratorias, Mexico City, Mexico; 8 Instituto de Investigaciones Biomédicas, Universidad Nacional Autónoma de México UNAM/Instituto Nacional de Cancerología, México City, Mexico; McMaster University, Canada

## Abstract

It is known that cancer progresses by vertical gene transfer, but this paradigm ignores that DNA circulates in higher organisms and that it is biologically active upon its uptake by recipient cells. Here we confirm previous observations on the ability of cell-free DNA to induce *in vitro* cell transformation and tumorigenesis by treating NIH3T3 recipient murine cells with serum of colon cancer patients and supernatant of SW480 human cancer cells. Cell transformation and tumorigenesis of recipient cells did not occur if serum and supernatants were depleted of DNA. It is also demonstrated that horizontal cancer progression mediated by circulating DNA occurs via its uptake by recipient cells in an *in vivo* model where immunocompetent rats subjected to colon carcinogenesis with 1,2-dimethylhydrazine had increased rate of colonic tumors when injected in the dorsum with human SW480 colon carcinoma cells as a source of circulating oncogenic DNA, which could be offset by treating these animals with DNAse I and proteases. Though the contribution of biologically active molecules other than DNA for this phenomenon to occur cannot be ruled out, our results support the fact that cancer cells emit into the circulation biologically active DNA to foster tumor progression. Further exploration of the horizontal tumor progression phenomenon mediated by circulating DNA is clearly needed to determine whether its manipulation could have a role in cancer therapy.

## Introduction

The current paradigm in cancer progression is that it occurs via vertical gene transfer; this means that the offspring of initiating tumor cell inherit the genetic and epigenetic alterations leading to tumor progression. This model, however, ignores that horizontal or lateral transfer of DNA connects and shapes nearly all living things [1] and that within an organism, circulating DNA, such as exosomes that contain transcriptionally active mRNA and microRNA, may potentially act as an endocrine or paracrine messenger, able to affect the functionality of recipients cells [2]. Accordingly, it has been proposed that cell-free DNA (circulating DNA) could participate in the development of metastases via “passive” transfection-like uptake of such nucleic acids by susceptible cells [3]. In 1994, Anker et al., first demonstrated that the supernatant of cultured colon cancer cell line SW480 was able to transform recipient immortal murine NIH3T3 cells, which acquire mutated human *K-ras* [4]. Transformation of these recipient cells by plasma of colon cancer patients has been reported as well [5].

The ability of genetic material to circulate in eukaryotes has been known since 1948 [6], and that this DNA can be released by bacteria and higher organisms and enter into recipient cells was demonstrated by Anker and Stroun [7]–[9]. These findings led to the concept that DNA could act as a messenger [10]–[16]. This view has been supported by the ease with which administered bacterial and eukaryote DNA can circulate freely throughout animal and plant bodies and in its ability to enter individual cells naturally, where it can locate in the host cell nuclei [12], [17]–[18]. The uptake of circulating DNA by eukaryotes shows that the biology of the recipient cells/organisms could be modified regardless of whether it is integrated or not [19]–[27].

It is noteworthy that such DNA administration does not require any special vehicles, e.g., liposomes, electroporation, and gene guns, to aid entry into cells in order for it to be biologically active. How genetic material circulates and transfers into somatic cells of higher organisms remains controversial. Although this is not mutually exclusive, some authors have demonstrated that this occurs via the uptake of apoptotic bodies [28], while others have characterized circulating DNA as a complex of DNA/RNA-lipoprotein termed “virtosome”, which is spontaneously released by living cells [29]. Here we demonstrate that circulating DNA is able to drive horizontal tumor progression in an immunocompetent colon-carcinogenesis rat model.

## Materials and Methods

### Cell Lines

SW480 (human colon cancer cell line that has the point mutation Gly to Val at codon 12 of exon 1 of the *K-ras* oncogene), HeLa (human cervical cancer cell line positive for HPV-18), and NIH3T3 (mouse immortalized fibroblasts) were purchased from the American Type Culture Collection. Primary culture of foreskin fibroblasts (BB1) was derived from circumcision foreskin of newborn boy under written consent from his father and used in the second passage.

### Supernatant Preparation

When cell cultures had a confluence of 80%, supernatants were collected by pipetting and cleared of any remaining cells and cell debris by a centrifugation step at 400×*g* for 20 min (Biofuge primo R, Heraeus) and passed through a 0.45-µm filter (Sartorius, 16555) to remove potentially contaminating cells. Aliquots of each supernatant samples were seeded in a culture flask and incubated at 37°C for 120 h to verify the absence of living cells. For DNA analysis, 50 ml of supernatant were concentrated, first with an ultrafiltration system with a 40 kDa membrane pore (Amicon, Stirred Ultrafiltration Cell, model 8010) and then with a speed vacuum device (Vacufuge Plus, Eppendorf) up to 10 ml. The concentrated supernatant was immediately cryopreserved at −80°C.

### Enzymatic Digestion of the Processed Supernatants

Fifty ml of the processed supernatants from SW480 (SW) and NIH3T3 (NH) were divided into 12.5 ml aliquots and incubated with proteinase K and/or DNAse I as follows: 1) SW+DNAse I; 2) SW+Proteinase K; 3) SW+DNAse I+Proteinase K, and 4) no enzyme. The control digestions were DMEM+2% FBS+salmon sperm DNA, and b) DMEM+2% FBS+salmon sperm DNA+DNAse I. Digestion was performed with 1.5 units (50 µg) of proteinase K per mg of total protein contained in supernatant for 60 min at 37°C followed by inactivation at 80°C for 20 min. For DNAse I, enzyme concentration was 12 units (3.6 Kunitz) per 12 µg of DNA content in the supernatant for 60 min at 37°C and then inactivation at 65°C for 15 min. Digestion with both enzymes was done with the same concentration and times but digesting first with proteinase K and then DNAse I. Following enzymatic digestion, the supernatant was used for “passive” transfection as indicated above. Degradation of proteins and/or DNA was verified by gel electrophoresis.

### Serum Collection and Preparation

Sera were extracted from blood of three patients with advanced colon cancer and healthy subjects. Blood was obtained from peripheral vein in two vacutainer tubes (Becton Dickinson, 368162) containing clot-activation additive and a barrier gel to isolate serum. The blood was kept at 4°C, and processed within 2 h, and centrifuged at 1000 *×g* for 20 min (Biofuge primo R, Heraeus) at room temperature; serum was collected and passed through a 0.45-µm filter (Sartorius, 16555) to remove cells. Blood samples were obtained with written consent from source patients.

### Passive Transfection of Mouse NIH3T3 Cells

NIH3T3 cells -as recipient for the transformation assays- were seeded in 24-well plates and exposed to human serum or supernatants SW480 (digested or not) in a 1∶1 proportion (DMEM with 2% FBS/serum or supernatant) for 14 days, refreshing the media every 24 hrs [4]. After 14 days of exposure (only seven days in plates exposed to serum), the cells were dispersed and propagated under standard conditions. Then the exposed cells were analyzed for the presence of mutated human *K-ras* sequences by PCR, RT-PCR, and sequencing. Experiments were performed by triplicates.

### Active Transfection (Lipofectamine)

One day before transfection, NIH3T3 cells were seeded at a density of 1×10^5^ cells per well (six-well plates) in 500 µl of growth medium without antibiotics (DMEM). Transfections were done using 5µg of DNA (either genomic DNA or supernatant DNA from SW480 cells plus 0.5 µg plasmid (pEGFP-CMV). Lipofectamine transfections were done following manufactureŕs instructions (Invitrogen™, LTX & Plus Reagent). Cells were incubated at 37°C for 24 h; medium was refreshed and the G418 selection was started to get stable transfectants.

### Transformation Assays

NIH3T3 that were passively transfected were employed to perform the transformation assays. As control, we used NIH3T3 cells exposed to the serum of a healthy donor and to their own supernatant. The characteristics used as indicators of malignant transformation were morphological changes (focus formation), anchorage-independent growth (growth in soft agar), and tumorigenicity [30].

### Morphological Analysis

Transfected NIH3T3 cells were examined for foci formation and counted under phase-contrast microscopy.

### Growth of Cells in Soft Agar

Foci were isolated from plastic culture plates and expanded for soft agar analysis. After trypsinitation cells were suspended in DMEM medium containing 0.3% noble agar and 15% FBS. A layer of this suspension was plated on top of a layer of medium containing 0.7% agar without serum. Cells were plated at a density of 5×10^5^ cells per 10-cm dish. Colonies was scored after 14 days of culture (a colony was defined as if contained at least 50 cells).

### In *in vivo* Experiments

Tumorigenesis assays were performed in 6-week-old athymic BALB/c (nu/nu) female mice (Harlan Laboratories). In all experiments, groups of six mice were injected subcutaneously with 2 million cells suspended in 100-µl of FBS-free DMEM. Tumor growth was monitored in and recorded weekly. Tumor size was measured with an electronic caliper and size-volume estimated using the formulae a×b^2^× (π/6) = V (mm^3^) (a = major diameter; b = minor diameter and V =  volume). In experiments to assess the tumorigenesis ability of supernatant of HeLa cells in unmanipulated animals, 6-week-old athymic BALB/c (nu/nu) female mice were also used. Animals were injected daily by intraperitoneal route with 500 µl of supernatant of HeLa cells for 30 days. At day 31, the animals were sacrificed. Athymic Hsd:RH-*Foxn1^rnu^* female rats and immunocompetent Hsd:Wistar female rats as well (Harlan Laboratories) were injected with apoptotic bodies from HeLa cells, which were obtained after high-dose exposure for 24 hours to cisplatin at 75 µM. Detached cells were harvested and centrifuged step at 400×*g* for 10 min (Biofuge primo R, Heraeus) in PBS three times to remove any remaining cisplatin. Injections of apoptotic bodies were done every other day by intravenous injection in the tail in a total volume of 100 µl of normal saline. Treatment lasted for 60 days. At day 61 rats were sacrificed and necropsy performed to macroscopically evaluate tumor formation. Major organs (liver, spleen, kidney, lung and uterus) were processed for H&E pathological examination.

To assess horizontal tumor progression, Hsd:Wistar 6-week-old female rats (Harlan Laboratories) were treated with either: i) no treatment; ii) subcutaneous injection of 1×10^6^ SW480 cells (diluted in 100 µl of normal saline) in the flank at days 28 and 49 of DMH treatment; iii) the colon carcinogen DMH by intraperitoneal route for 20 weeks as reported [31]; iv) the regimen of DMH plus SW480 cells; and v) as group iv plus DNAse I/protease treatment which consisted on DNAse I at a dose of 2.3 mg/Kg by intramuscular injection and a mix of proteases (trypsin, chymotrypsin, and papain) by intraperitoneal injection at doses of 10 mg/Kg, 10 mg/Kg and 25 mg/Kg respectively, as reported in [32]. Both DNAse I and proteases mix were diluted in 100 µl of normal saline. Injections were administered daily except weekends from week 4 to 12. Group vi received DMH+DNAse I/proteases. After evaluation with micro PET-CT (as described below) animals were sacrificed and autopsied 6 months after finishing the 20 weeks of carcinogen (DMH).

To evaluate the fate of human SW480 cells injected into immunocompetent animals Hsd:Wistar 6-week-old female rats (Harlan Laboratories) were subcutaneously injected with 10-million SW480 cells in the flank and sacrificed at days 1, 2, 3, 7, and 14 after injection to remove the injection site and processed for routine H&E histological analysis. Ethical approvals were obtained from the Institutional Research Ethics Board and Animal Care Committee.

### Micro PET-CT

Tumor formation in the animals was monitored using molecular imaging techniques with a micro PET-CT (Albira ARS, Oncovision) and ^18^F-FDG. Tumor monitoring was conducted at weeks 15 and 24 after the start of treatment (DMH). To quantify tumor activity, the standard uptake value (SUV) was calculated utilizing Albira system software tools (Carestream Molecular Imaging, CT, USA). SUV is a quantitative tool for PET-CT studies that allows for determination of the ^18^F-FDG concentration in a specific region-of-interest (i.e., tumor activity). Tumor presence was defined when the SUV (tumor/liver) ratio was ≥1.5.

### Tissue Preparation and Microdissection

The formalin-fixed, paraffin-embedded colonic tumors (one of each group) from each group of DMH-treated rats were cut into 5-µm-thick sections on a microtome with a disposable blade. For microdissection, sections were deparaffinized in two changes of xylene for 10 min, rehydrated in 100% ethanol, 90% ethanol, and 70% ethanol for 5 min each, stained with hematoxylin and eosin (H&E) for 45 sec, rinsed in RNase-free H_2_O for 30 sec, and finally immersed in 100% ethanol for 1 min. The PALM Laser-MicroBeam System (P.A.L.M., Wolfratshausen, Germany) was used for microdissection. After selecting the cells-of-interest, adjacent cells were photolysed by the microbeam. To retrieve the selected cells from the slide, a computer-controlled micromanipulator and conventional sterile needles were used to pick up and transfer the cells into a reaction tube.

### DNA Extraction

Circulating DNA extraction was performed by SDS/proteinase K digestion followed by phenol/chloroform extraction as described by Anker, P [4]. Briefly, 500 µl of serum or supernatant were mixed with 500 µl of a solution of SDS/proteinase K (Invitrogen) and incubated overnight at 55°C. An equal volume of phenol/chloroform (1∶1 v/v) was added, vortexed briefly, and centrifuged at 800×g for 10 min (Biofuge primo R, Heraeus). The aqueous phase was recovered and mixed with an equal volume of chloroform and centrifuged at 800 x g (Biofuge primo R, Heraeus) for 5 min. The aqueous phase was precipitated overnight at −20°C with 1/10 volume of 7.5 M ammonium acetate, 1 µl of glycogen, and 2.5 volumes of 100% ethanol and then centrifuged at 1200×g (Biofuge primo R, Heraeus) for 45 min. The DNA pellet was washed with 70% ethanol, air-dried, and resuspended in water. Quantification of total DNA was performed using the PicoGreen assay (Invitrogen) following the manufacturer’s instructions. DNA from microdissected tumors of paraffin-embedded tissue was extracted with the PicoPure™ DNA isolation kit (ARCTURUS Mountain View CA) following manufactureŕs instructions.

### RNA Extraction

Total RNA was isolated from processed supernatant and human serum using QIAamp Viral RNA Mini Kit (Qiagen, Hilden, Germany), following the manufacturer’s instructions.

### PCR

All reactions were performed in 20 µl containing 100 ng of template DNA, 10 mmol/l Tris-HCl (pH 8.3), 40 mmol/l KCl, 2 mmol/l MgCl_2_, (1 mmol/l MgCl_2_ for *RAB30*, and 5 mmol/l MgCl_2_ for *Alu Yd6*), 200 µmol/l of each dNTP, 0.25 U Taq polymerase (Applied Biosystems), and 1 µmol/l of each specific primer: human *K-ras* (5′-gactgaatataaacttgtggtagt-3′, and 3′-ggacgaatatgatccaacaatag-5′), 107 bp amplicon; *E6* (5′ gggggatccatggcgcgctttgaagatccaaca-3′, and 3′-ggggaattcttatacttgtgtttctctgcgtcg-5′), 450 bp amplicon; *E7* (5′-cccgacgagccgaaccacaac-3′, and 3′-gggatgcacaccacggacacac-5′), 300 bp amplicon; human *DHFR* (5′-agaaccaccacgaggagc-3′, and 3′-acagaactgcctccgactatc-5′), 120 bp amplicon; human *ACTIN* (5′-ggagtcctgtggcatccacg-3′, and 3′-ctagaagcatttgcggtgga-5′), 320 bp amplicon. Human *RAB30* (5′-gtccattacccagagttactaccg-3′, and 3′-gaccttgttgctggcatattgttc-5′), 130 bp amplicon; *Alu Yd6* (5′-gagatcgagaccacggtgaaa-3′, and 3′-ttgctctgaggcagagttt-5′), 200 bp amplicon [33].

An initial denaturation at 94°C for 5 min was followed by 40 cycles of amplification and a final extension step 5 min at 72°C, (*E6*, *E7* and *Alu Yd6* had extension time for 30 sec). The cycles included denaturation at 94°C for 30 sec, 30 sec of annealing (60°C for *K-ras* and *RAB30*, 59°C for *ACTIN* and *DHFR*, 57°C for *E6* and *E7* and 61°C for *Alu Yd6*), PCR reactions were carried out in a 2400 Thermalcycler (Applied Biosystems). The amplification products were verified by agarose gel electrophoresis. For rat specific *LINE 1* sequences the amplification was performed in 20 µl reactions containing 100 ng of template DNA, 10 mmol/L Tris-HCl (pH 8.3), 40 mmol/L KCl, 1 mmol/L MgCl_2_, 200 µmol/L of each dNTP, 0.25 U Taq polymerase (Applied Biosystems) and 1 µmol/L of each primer specific for rat *LINE 1* (5′-aaatcagggactagacaaggctgc-3′, and 3′-cccagccactttgctgaagttgt-5′) [34]. Initial denaturation at 94°C for 5 minutes was followed by 40 cycles of amplification and a final extension step (5 minutes at 72°C). Amplification cycles consisted on denaturation at 94°C for 30 seconds, annealing at 59°C for 30 seconds, and extension at 72°C for 30 seconds. PCR reaction was carried out on a 2400 Thermalcycler (Applied Biosystems). The amplification products were verified by agarose gel electrophoresis.

### RT-PCR

For first-strand cDNA synthesis 1–5 µg of total RNA was reverse-transcribed in a 20-µl reaction volume containing 2 µl of 10× PCR buffer, 50 ng of random hexamers, 50 mM MgCl_2_, 200 ng nM dNTP, 0.1 M DTT, and 200U Reverse Transcriptase, (GeneAmp, Applied Biosystems) for 50 min at 42°C. PCR amplification of cDNA was performed in a 20 µl reaction volume containing 100 ng cDNA, 10 mmol/L Tris-HCl (pH 8.3), 40 mmol/L KCl, 1 mmol/L MgCl_2_ (for *K-ras* ^v12^ and *RAB30*), and 200 µmol/L of each dNTP, 0.25 U Taq polymerase (Applied Biosystems), and 1 µmol/l of each primer specific for human *K-ras* ^v12^ (5′-actgaatataaacttgtggtagttggacct-3′, and 3′-caaatcacatttatttcctaccaggacct-5′), and for *RAB30* (5′- gtccattacccagagttactaccg-3′, and 3′-gaccttgttgctggcatattgttc-5′). Initial denaturation at 94°C for 5 min was followed by 40 cycles of amplification and a final extension step (5 min at 72°C). The cycles included denaturation at 94°C for 30 sec, annealing at 58°C (*K-ras* ^v12^), and at 56°C (*RAB30*) for 30 sec, and extension at 72°C for 30 sec. The size of the amplicons and the sequence of the primers used for PCR for *K-ras* ^v12^ is 357 bp, and for *RAB30*, 130 bp. The PCR reaction was carried out on a 2400 Thermalcycler (Applied Biosystems). The amplification products were subjected to electrophoresis on a 3% agarose gel.

### DNA Sequencing

To verify the origin of the amplified sequence (human or murine), the PCR products were sequenced. PCR amplicons were purified using isopropanol precipitation and then sequenced in both forward and reverse directions from at least two independent amplification products. Purified DNA was diluted and cycle-sequenced using the ABI BigDye Terminator kit v3.1 (ABI, Foster City, CA, USA) according to manufacturer’s instructions. Sequencing reactions were electrophoresed in an ABI3100 genetic analyzer. Electropherograms were analyzed in both sense and antisense directions. The sequences obtained were compared with the reference *KRas* sequence (GenBank DQ893829) and the *RAB30* sequence (GenBank NM_014488).

### Southern Blot

Labeled SW480 genomic DNA was hybridized to the following cell lines: SW480 (positive control); NIH3T3 (negative control), and a pool of *K-ras* positive cell clones derived from NIH3T3 exposed to human serum from a patient with colon cancer. Ten µg of high-molecular-weight genomic DNA of each DNA sample was digested with *Hind III* (New England Biolabs). The DNA fragments were separated by electrophoresis in a 0.8% agarose gel, denatured, and transferred onto nylon membrane (Amersham). Hybridization was performed in a solution of the BM Chemiluminiscence Blotting kit (Roche Applied Science) containing the DNA probe (100 ng/ml) for 20 h at 42°C. The blots were washed under high-stringency conditions in 0.1×SSC, 0.1% SDS for 90 min at 65°C, incubated in streptavidin for 30 min at room temperature, placed on Hyperfilm ECL (GE Healthcare Life Sciences), and exposed for 60 sec.

### SNP Array Hybridization and DNA Copy Number Analysis

DNA from parental SW480 cells, DNA isolated from supernatant of SW480 cells, and genomic DNA from SB1 cells (NIH3T3 passively transformed by SW480 supernatant) was hybridized onto the 500K Affymetrix genotyping array set for DNA copy number analysis, following the manufacturers protocol. Analysis was performed using the DNA copy number pipeline in the Partek Genomics Suite software. All samples were compared against a common normal human DNA copy number reference baseline.

### FISH

Interphase nuclei and metaphase chromosomes of the transfected NIH3T3 cells (SB1) were analyzed in standard cytogenetic preparations, as previously described [35], with a consensus sequence of human interspersed repeats. The human Cot DNA probe (which is placental DNA that is predominantly 50–300 bp in size and enriched for repetitive DNA sequences such as *Alu* and *Kpn* family members) was labeled by nick translation with digoxigenin-11-dUTP (Roche) by using the DIG-Nick Translation Mix (Roche) and detected utilizing a fluorochrome-conjugated antidigoxigenin antibody. One hundred nuclei were microscopically analyzed.

FISH analysis of histological sections of colon tumors of rats was done as follows: Sections were deparaffinized in xylene and rehydrated in graded ethanol series. The slides were pre-treated with 0.2 M HCl, 8% sodium thiocyanate, and 0.5% pepsin. Afterward, red-fluorescein-labelled *ALU* human (FISHBright, Kreatech Biotechnology) and green-fluorescein-labelled *LINE 1* rat (FISHBright, Kreatech Biotechnology) probes were added simultaneously; the slides covered with coverslips and sealed with rubber cement. Sections were denatured and hybridized in a Hybridizer (Life Technologies) at 37°C overnight. The rubber cement and the coverslips were removed and the sections were washed stringently using SSC: 2×SSC for 30 min at room temperature, 0.4×SSC/0.3% NP40 for 5 min at 75°C, and 2×SSC/0.1% NP40 for 4 min at room temperature. Nuclei were counterstained using 4′,6′-Diamino-2-phenylindole (DAPI) at a concentration of 0.1-µg/ml in Antifade (Vector Laboratories). Analyses were performed using a Zeiss Axioplan fluorescence microscope (Zeiss) interfaced with the CytoVision system (Applied Imaging).

## Results

### Extracellular DNA Transforms Immortalized Murine Cells Associated with DNA Transfer

**Figure 1:**
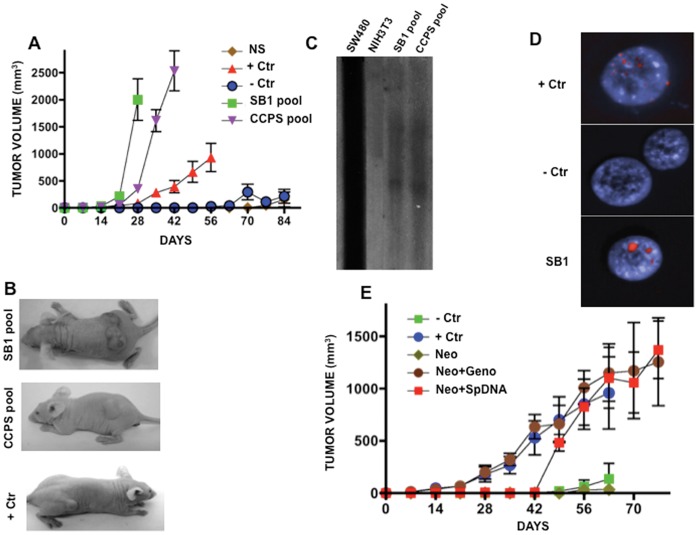
Tumorigenesis and DNA transfer in recipient murine NIH3T3 cells after passive transfection. A. Tumor growth in nude mice from “passively” transformed cells. Faster and higher tumor growth was observed in SB1 pool and CCPS pool (NIH3T3 exposed to supernatant of SW480 cells and to the serum of a patient with colon cancer, respectively). SW480 cells were used as positive control, whereas NIH3T3 and NIH3T3 exposed to normal serum showed essentially no growth**. B.** Representative pictures of tumors in mice from each group. **C.** Southern blot hybridization of SB1 and CCPS pools of cells against genomic DNA of SW480 cells. Lane SW480 cells are the positive control and NIH3T3, the negative one. A clear hybridization signal is only observed in SB1 and CCPS lanes. **D.** FISH analysis of repetitive human sequences. Positive control is human lymphocytes and murine cells negative control. SB1 cells shows strong signal. **E.** Tumor growth is similar in NIH3T3 actively transfected with genomic DNA from SW480 cells (Neo-Geno) and actively transfected DNA extracted from supernatant of SW480 cells as compared with no growth in NIH3T3 (-Crt) and transfected with the empty-vector only. Positive control, SW480 cells.

Cell-culture supernatants of human malignant cell lines SW480 and HeLa (grown in DMEM with 2% FBS), as well as serum of the three advanced untreated colon cancer patients and two healthy controls were cleared of viable cells and cell debris by centrifugation and filtering (0.45-µm filter). The DNA extracted from these sources, (SW480 and HeLa supernatants) were PCR-positive for mutant human *K-ras* at codon 12, and *E6* and *E7* oncogenes respectively. The three serum samples for cancer colon patients harbored the *K-ras* mutation at codon 12, which also was proven in DNA extracted from their paraffin-embedded primary tumors. These material (supernatants and serum of colon cancer patients and healthy controls were PCR positive for constitutive human *DHFR* and *ACTIN* genes (not shown). Serum DNA concentrations in serum were 2.04, 0.66 and 0.93 µg/ml for patients and 0.28 and 0.178 µg/ml for healthy individuals. Supernatants from five independent cell cultures of SW480 which were taken at 80% cell confluence, had a mean concentration of 0.1875±0.007 µg/ml. “Pasive” transfections with supernatants or sera were done as follows: 0.5 ml (serum or supernatant containing 1.815 µg or 0.09 µg respectively) were added to 0.5 ml of medium (1∶1 ratio) of cultured recipient murine immortal cells NIH3T3 (which were proven murine by cytogenetic analysis and by the absence of repetitive human *Alu Yd6* by PCR, not shown) in 24-well microtiter plates. Medium plus serum or supernatant were changed daily for 7 or 14 days (shorter time for serum due to the limited amount), thus, cells were exposed daily to 1.815 µg of DNA contained in the serum or to 0.09 µg of DNA contained in the supernatant DNA. At day 8 or 15, the number of transformation foci of recipient NIH3T3 cells in plastic and colony formation in agar were overwhelmingly superior for both serum and supernatant (as compared with the control (NIH3T3 cells exposed to their own supernatant and to normal serum). Among established transformed clones, 11 of 27 exposed to SW480 and 5/9 (exposed to the serum of the patient with cancer) had the *K-ras* mutation as proven by PCR utilizing primers specific for amplifying human *K-ras*. Likewise, 8/24 clones exposed to HeLa were PCR-positive for *E6* and *E7* HPV oncogenes. Tumorigenesis assays in *nu/nu* female mice showed larger and faster growing tumors from a pool of clones “passively” transfected with supernatant of SW480 (SB1 pool) and colon-cancer serum (CCPS) as compared with parental SW480 cells (+Ctr), with murine NIH3T3 exposed to normal serum (NS), as well as with parental NIH3T3 (−Ctr) (**Fig. 1** **A, B**). These results confirm previous observations on the transforming ability of the supernatant of SW480 cells, as well as those of plasma from patients with cancer [4], [5]. Southern hybridization of these NIH3T3-transformed pools of clones (SB1 pool) against genomic DNA from SW480 cells showed clear hybridization signals, confirming the horizontal transfer of human DNA to recipient murine cells (**Fig. 1** **C**). Further, FISH analysis of passively transformed murine cells (SB1) emitted positive signals for human repetitive sequences (**Fig. 1** **D**), which confirms that cell transformation was associated with DNA transfer.

To demonstrate that the transforming ability of the supernatant resides in the DNA, 5µg of purified DNA from the SW480 supernatant was co-transfected with a eukaryotic pNeo vector utilizing lipofectamine (active transfection). Multiple genetecin-resistant clones were established. A pool of these clones was able to form tumors in *nu/nu* female mice (**Fig. 1** **E**). Contrariwise, “passive” transfection using 5 µg of purified DNA (extracted from cell-culture supernatant) and added daily for 14 days to NIH3T3 cells) was unable to transform NIH3T3 cells and also failed to transfer DNA sequences into recipient cells (as evaluated by PCR of mutant human *K-ras* and *Alu Yd6*, not shown).

### DNA-depleted Supernatant does not Transform Immortalized Murine Cells

**Figure 2:**
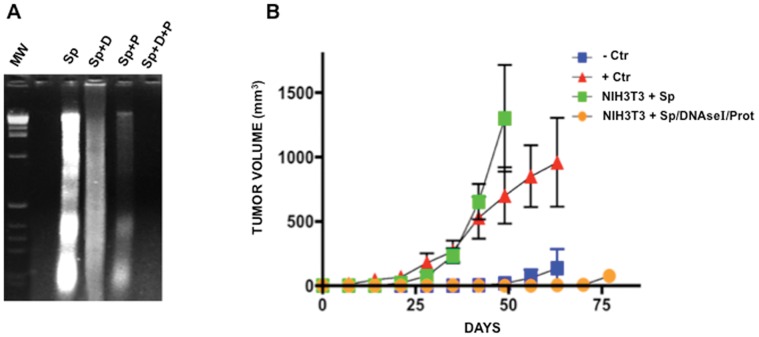
Tumorigenesis after “active” transfection with DNA supernatant of SW480 cells and passive transfection using DNA-depleted supernatant. A. Agarose gel electrophoresis of DNA extracted from supernatant (Sp), DNA from supernatant treated with DNAse I (Sp+D), protease only (Sp+P), and both (Sp+D+P). DNA is partially degraded by DNAse I and protease, but fully degraded when exposed to both treatments. **B.** No tumor growth was observed in NIH3T3 exposed to DNA-depleted (DNAse I/Prot) supernatant of SW480 cells, while passively transfected NIH3T3 with untreated supernatant are tumorigenic. +Ctr are SW480 cells and −Ctr are NIH3T3 cells.

It has been shown that cell-free DNA circulates as a complex of the DNA/RNA-lipoprotein (virtosome) that is spontaneously released by living cells [29] and this complex could protect DNA from being degraded by circulating nucleases. To investigate this, fresh supernatant of SW480 cells was exposed to DNAse I, to proteinase K, and to both, and then the DNA was extracted and electrophoresed. Results showed that DNAse I failed to digest DNA fully. Likewise, exposure of supernatant to proteinase K only mildly digested DNA. Contrariwise, exposure first to proteinase K and then to DNAse I completely degraded the DNA (**Fig. 2** **A**). This finding allowed us to probe further that the supernatant DNA is responsible for transformation as “passive” transfection with DNA-depleted supernatant (treated with both enzymes) on NIH3T3 cells failed to induce focus formation, growth in agar, and tumorigenesis in nude mice (**Fig. 2** **B**)**.** In summary, these results show that the transforming ability of the supernatant of SW480 cells resides in the DNA.

### Gene Copy Number is Nearly Identical between Intracellular and Extracellular DNA Compartments

**Figure 3:**
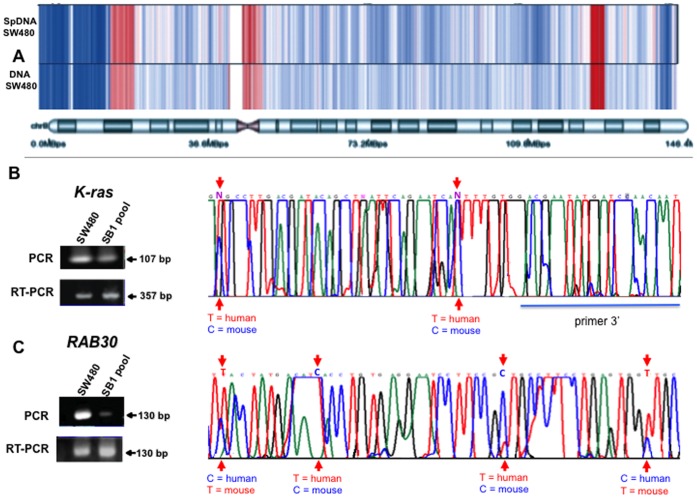
DNA copy number analysis of extracellular and intracellular DNA from SW480 cells and gene transfer to murine NIH3T3 cells. A. Heat map representing the DNA copy number along chromosome 8. Blue represents regions with deletions and red regions with amplifications. A nearly identical pattern of DNA copy number changes between extracellular (SpDNA SW480) and intracellular (DNA SW480) DNA, compared to a common normal reference can be observed. RT-PCR, PCR and sequencing of Human *K-ras* (**B**) and *RAB30* (**C**)**.** Negative control was NIH3T3 cells.

Sequencing analyses, indicate that circulating DNA comes from the whole genome, with minimal indications of sequence clustering, although there is incomplete coverage of all chromosomes [36]. In contrast, unequal distributions of genes have been described [37]. In any case, the results of our Southern hybridization experiments (**Fig. 1** **C**) at the very least suggest that transfer may not be limited to a few DNA sequences. To gain further insight into this, we hybridized genomic DNA (intracellular) from parental SW480 cells, DNA isolated from supernatant of SW480 cells (extracellular), and genomic DNA from SB1 cells (NIH3T3 passively transformed by SW480 supernatant) onto the 500 K Affymetrix genotyping array set for DNA copy number analysis. All samples were compared against a common, normal human DNA copy number-reference baseline. High-quality hybridization was obtained with the SW480 genomic DNA (intracellular), as well as with the DNA obtained from the SW480 supernatant (extracellular). Results showed that there is a nearly identical pattern of DNA copy number profile between the “intracellular” and “extracellular” DNA of SW480 cells as profiled in **Fig. 3** **A** showing chromosome 8, and **Suppl.** **Fig. S1** showing all chromosomes. DNA (intracellular) from SB1 cells was hybridized as a potential way to acquire human DNA genes transferred into the NHI3T3 cell genome; however, the overall quality of the hybridization using this DNA was poor and we were unable to obtain any clear signal that might suggest that recipient cells possess preferential uptake of the specific genomic regions present in the supernatant DNA of SW480 cells (GEO accession number GSE35052). To confirm the copy number analysis data, we selected the human *K-ras* and *RAB30* genes, which were found amplified and single copy respectively in both DNAs (intracellular and extracellular). These genes were PCR- and RT-PCR-amplified using human-specific primers followed by sequencing in SB1 cells (**Fig. 3**) illustrates that for both genes (**B** *K-ras*, **C** *RAB30*), mice and human bases were detected in the mRNA sequences, confirming, at least for these two genes, that regardless of whether the gene in supernatant is amplified or not, recipient cells can acquire these from the supernatant.

### Extracellular DNA Fails to Transform Primary Human Cells

NIH3T3 cells are embryonic murine fibroblasts, which are immortalized and prone to spontaneous transformation; hence, we wanted to investigate whether the supernatant DNA was able to transform early-passage primary human-foreskin fibroblasts (BB1). Exposure of these primary cells to SW480 supernatant even for 45 days failed to induce foci formation, and when injected into *nu/nu* mice exhibited no tumorigenesis at all (**Suppl.** **Fig. S2A**). The inability of extracellular DNA to transform primary cells was also confirmed *in vivo*. Six *nu/nu* mice received daily intraperitoneal (i.p.) injection of HeLa supernatant for 30 days. No tumors were clinically observed and a comprehensive autopsy found no histological alterations in the organs examined (lung, liver, spleen, colon, and kidney), supporting the *in vitro* observations. Further, six female *nu/nu* rats and six immunocompetent Wistar rats were intravenously injected every other day with apoptotic bodies obtained from HeLa cells for two months and animals sacrificed after finishing injections. Again, no clinical alterations were observed and no dysplastic or neoplastic cells were found in these organs on histological analysis. To rule out that the lack of tumorigenesis was due to the non-occurrence of DNA transfer, liver, spleen, colon, uterus, lung, and kidney of these animals were examined for the presence of *E6* and *E7* oncogenes both at the genomic and the expression level as evaluated by PCR and RT-PCR. The results showed that both *E6* and *E7* were detected by PCR while *E7* expressed, although only in liver (**Suppl.** **Fig. S2 B**).

### Transfer of Extracellular DNA and Tumor Progression in Rats

All of these data indicated that, most probably, lateral transfer of oncogenes by extracellular DNA is insufficient to induce tumor formation in primary normal cells and in unmanipulated animals (either immunodeficient or -competent), and that perhaps an *initiating* event was required to develop malignant tumors in line with the current model of carcinogenesis -*initiation-promotion-progression*. To prove this, immunocompetent female Wistar rats were treated with the colon carcinogen 1,2-Dimethylhydrazine (DMH) by i.p. route for 20 weeks (20 mg/kg of DMH each week). At week 4, rats were injected s.c. (twice, 15 days apart) with 10 million of SW480 colon-cancer cells in the flank. The experimental scheme is depicted in **Suppl. Fig. S3**. The groups were treated as follows: i) control (no DMH, no SW480 injection, 7 rats); ii) SW480 cell injection only (7 rats); iii) DMH only (12 rats), iv) DMH+SW480 cells injection (16 rats), v) DMH+SW480 injection plus DNAse I/proteases, 6 rats and vi) DMH plus DNAse I/proteases, 5 rats.

**Figure 4:**
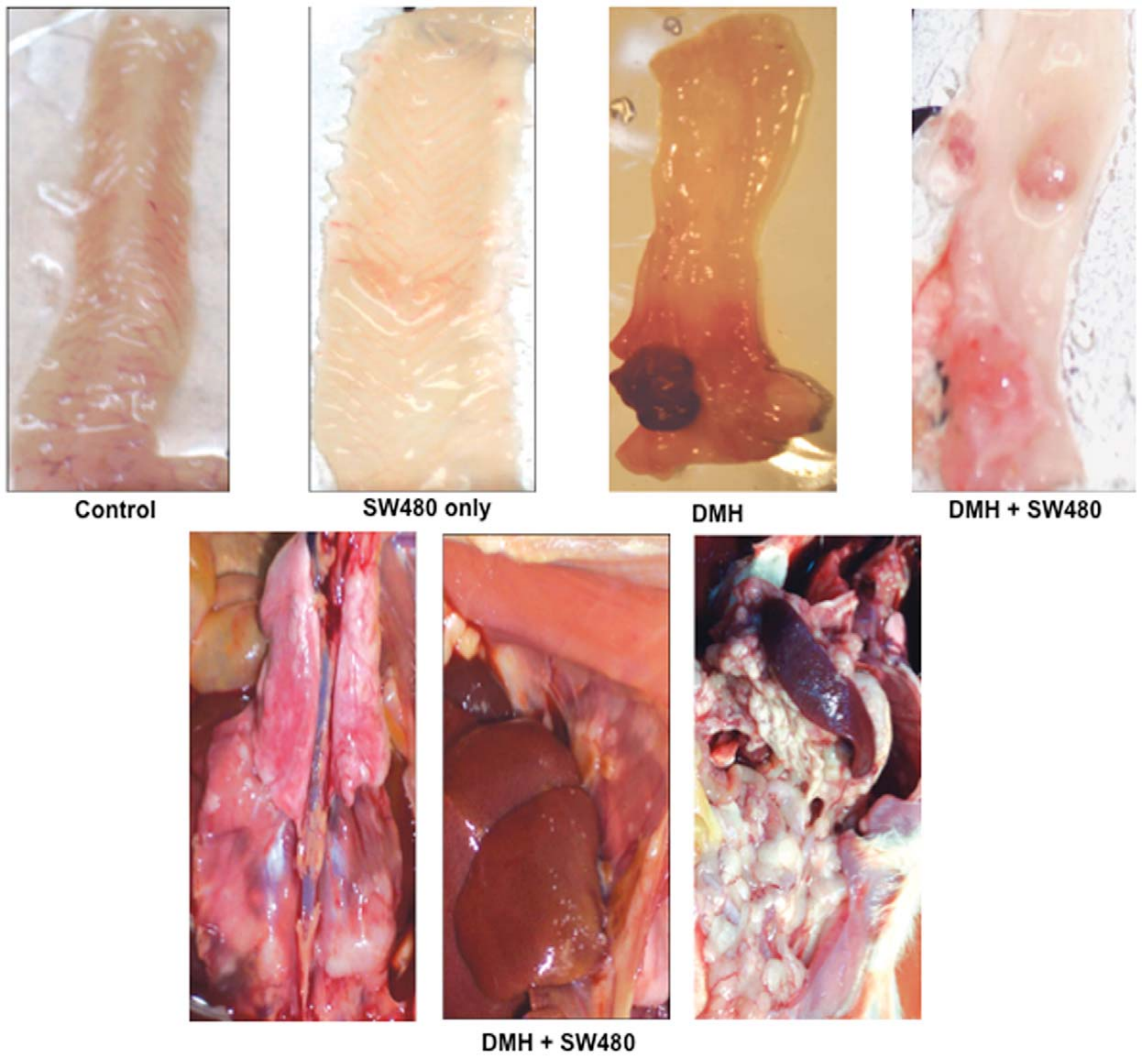
Tumorigenesis in the immunocompetent Wistar rat model. Macroscopic aspect of colon in rats. Control rats and those only receiving SW480 cells had no tumor formation. A colon tumor is observed in the rat treated with DMH, (group iii), whereas 3 tumors are observed in a rat receiving both DMH and SW480 cells (group iv). The inferior panel shows representative pictures of a rat from group iv (DMH+SW480 cells) showing extensive peritoneal carcinomatosis.

After 15 weeks of starting treatment with DMH and 1 month after finishing the 20 weeks of carcinogen (DMH) administration, 2 rats from each group were evaluated for tumor formation with micro PET-CT (Albira ARS) studies with ^18^F-FDG using the SUV ratio (Tumor/Liver). Tumor presence was defined when the SUV ratio was ≥1.5. Results showed that rats from groups (i), (ii) and (v) had no tumors; one of two animals receiving DMH (group iii) had a tumor detected (**Suppl. Fig. S4 A**), whereas two of two from group (iv) had tumors (**Suppl.** **Fig. S4 B**). The initial micro PET-CT evaluation in the rat from group (iii) with tumor had a SUV ratio of 2.7, and 2.2 at the second evaluation. The respective SUV ratios for the two rats of group (iv) were 1∶6 and 1∶51 in one rat, and 3∶9 and 4∶1 for the second rat. Then, the rats were sacrificed and a comprehensive autopsy performed. Macroscopic and histological evaluation of animals showed that two of 12 (17%) rats from group (iii) had tumors. In these cases, no extra-colonic dissemination was observed. In group (iv) rats that received DMH and injection of SW480 cells, 10 of 16 (62.5%) had tumors (two-tailed Fisher exact test, *p*  = 0.0235). Interestingly, only one out of 6 rats from group (v) which were treated like group iv plus DNAseI/protease had tumors (16.6%) which are similar to rats treated only with DMH (group iii) and proportionally inferior to group (iv) 62.5% though this difference was not statistically significant (p = 0.144). Two out of 5 (40%) rats from group (vi) DMH+DNAse I/protease treatment had tumors. (**Table 1**). Three animals from group iv presented extensive peritoneal (accompanied by ascites) and pleural dissemination of tumor cells (representative pictures are shown in **Fig. 4**). Macroscopic colonic tumors were counted in these groups. Group (iii) had a mean of 1.5±0.70 tumors, whereas in group iv, this number was 3.85±1.2 tumors (unpaired Student *t* test, *p*  = 0.0305; [95% CI]: −4.30 to −0.270).

**Table 1:** Results from the *in vivo* tumorigenesis en the immunocompetent Fisher rats.

Group	Treatment	Animals	Tumors (%)
**(i)**	none	6	0 (0)
**(ii)**	SW480 cells injection	7	0 (0)
**(iii)**	DMH	12	2 (16.6)
**(iv)**	DMH+SW480 cells injection	16	10 (62.5)*
**(v)**	DMH+SW480+D/P	6	1 (16.6)
**(vi)**	DMH+D/P	5	2 (40%)

D/P means DNAseI/protease treatment.

* Statistically significant with the Fisher exact test as compared to group (iii).

Neither control rats nor those receiving only SW480 cell injection had macroscopic or microscopic evidence of tumors. To rule out that colon tumors in these rats originated from metastatic SW480 cells injected in the flank, another group of rats (two animals each time point) were injected in the flank with the same amount of SW480 cells used in the DMH experiments and the animals were sacrificed at days 1, 2, 3, 7, and 14 after injection; the injection site was removed and processed for histological analysis. The results showed a decreasing amount of viable cells at days 1, 2, 3, and 7, but viable cells were not found at day 14. It is noteworthy that a progressive inflammatory infiltrate and apoptosis were found at these time points (**Suppl. Fig. S5**). These results were not unexpected, because immunocompetent rats reject malignant human SW480 cells, ruling out that colon tumors from DMH-treated and SW480-injected cells, originated from viable, metastatic, SW480 human cells.

**Figure 5:**
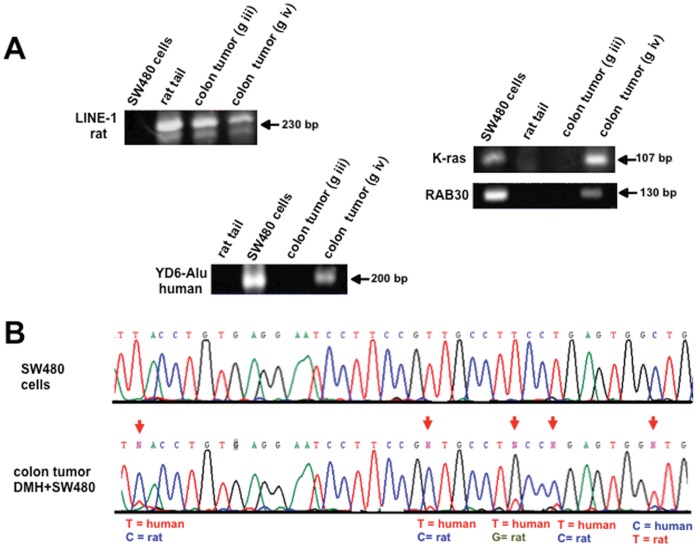
Human DNA transfer in rat colon tumors by PCR-sequencing. Representative pictures of PCR detection of a repetitive sequence of rat (*LINE 1*) in a rat tumor (DMH and DMH+SW480). (**A**) *Alu Yd6* human sequences were only amplified from the colon tumors of DMH+SW480-treated rats. Rat tail and human cells were used as positive and negative controls. Human *K-ras* and *RAB30* genes were only detected in the tumors of rats receiving DMH and SW480 cells. (**B**) Sequence analysis of the PCR product of *RAB30* in a colon tumor treated with DMH+SW480 cells. Arrows indicate the position where the nucleotide sequence is different between species and clearly shows the existence of both sequences. Human SW480 cells (control).

**Figure 6:**
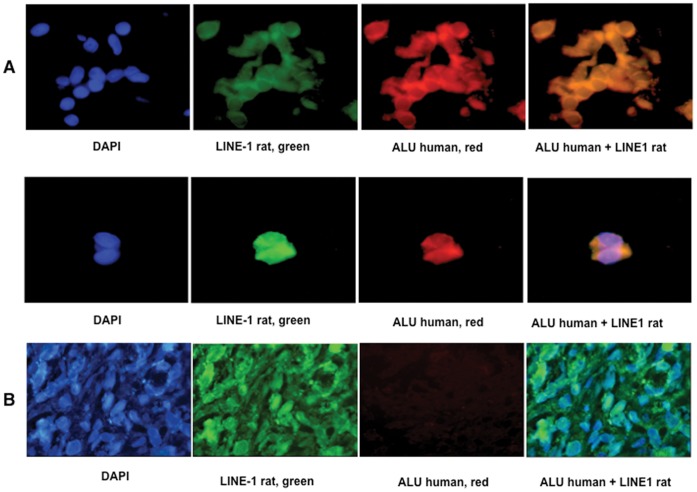
Human DNA transfer in rat colon tumors by FISH. Representative photographs FISH analyses of rat repetitive sequences (*LINE 1*) and human (*Alu Yd6*) in a colon tumor from a rat treated with DMH+SW480 cells (**A**)**.** Pictures at left (blue) are nuclei stained with DAPI, green are rat specific *LINE 1* signals. Red are human *Alu Yd6* signals and orange are cells showing both signals. **B**. Representative photographs of a colon tumor from a rat treated with DMH only. Absent red signals represent the lack of human sequences in these cells. Co-incubation with both probes at the right confirms the lack of DNA transfer.

A colonic tumor from each group of rats was microdissected and DNA was extracted from tumor cells (DNA from SW480 human cells and rat tail were used as controls for PCR reactions). All tumors were positive for rat *LINE 1* sequences, but *ALU* human sequences were positive only in the tumors of rats that received DMH plus SW480 injection (group iv) as also were positive for human mutant *K-ras* and human *RAB30*. **Fig. 5** (**A**)**.** Of note, we were not able to detect either of these human sequences in the tumor of the rat from group (v) which received DNAse I/protease treatment. Sequencing of these amplicons showed rat and human bases in the sequences as shown in **Fig. 6** (**B**) for the *RAB30* gene. The transfer of human DNA to rat colonic tumors was also evaluated by FISH analysis in paraffin-embedded tumors (**Fig. 6**). Colon tumor cells from a DMH-treated and SW480-injected rat (group iv) were FISH-positive for both *ALU* human and rat repetitive sequences (*LINE 1*) (**A**), whereas rat tumors from the DMH-only group and from group (v) were negative for human, but positive for rat sequences (**B**)**.** These results confirmed that circulating DNA-induced transformation also occurs *in vivo* and that it accounted for tumor progression.

## Discussion

The results of this study show that extracellular DNA does not only induces cell transformation and tumorigenesis but also drives tumor progression *in vivo*, establishing that cancer progression also occurs by horizontal or lateral DNA transfer in this model.

Our results support that circulating DNA behaves as a signaling endocrine molecule that contributes to cancer progression. This is based primarily on two facts; i) tumor cells shed DNA into the circulation of cancer patients [38], [39] and ii) circulating DNA has the ability to enter individual cells [12], [17]–[18] and it can modify the biology of the recipient cells/organisms regardless of whether it is integrated or not [19]–[27]. In line with these data and our results, we hypothesize that in cancer patients the tumor DNA shed into the circulation is inserted into “normal-appearing” initiated stem cells, which are transformed by the circulating DNA, leading to “second primary tumors” (**Suppl.** **Fig. S6**). In this scenario, although “second primary tumors” can clinically be considered metastatic tumors, would, in fact, originate via horizontal DNA transfer, and this concept fits well into the in-field carcinogenesis concept, in which multiple “initiated” cells are prone to transformation. It also can be hypothesized that circulating DNA could be a trigger for tumor progression according to the currently proven metastatic progression concept. In this scenario, as it is known, primary tumors disseminate into distant organs by the process known as micrometastases at a very early stage, even prior to being invasive [40]. Micrometastatic cells then could acquire DNA from the primary tumor via horizontal DNA transfer, which would induce these micrometastases to grow and to form metastatic lesions as clinically defined. In any case, DNA-induced cell transformation and tumor progression could possibly be aided by the concurrent transfer of one or several biologically active molecules or humoral factors, known to be present in particles shed into the circulation by cancer cells [41].

Other studies on horizontal DNA-induced cell transformation using different models came in general, to the same conclusions on the ability of exogenous tumor DNA to induce cell transformation and/or tumorigenesis. By using supernatant of cultured SW480 cells Anker et al., showed NIH3T3 transformation associated with mutant *K-Ras* transfer [4]. Garcia-Olmo et al, observed both cell transformation and tumorigenesis of NIH3T3 “passively” transfected with human plasma of colon cancer patients. In addition, they showed that plasma from healthy individuals was unable to do so, and that plasma of colon cancer patients failed to induce transformation of human adipose-derived stem cells obtained from lipoaspirates of non-cancer patients [5]. Similar results have been obtained using apoptotic bodies as a source of exogenous DNA. Bergsmedh et al., used *H-ras*/human *c-myc*-transfected rat fibroblasts as donor and mouse embryonic fibroblasts as recipient cells [28] while Gaiffe et al., [42] used human HPV-positive cervical cancer cells and human mesenchymal cells taken from an adult human after abdominoplasty as source and recipient cells respectively. Interestingly, they were able to show cell transformation in these recipient non-immortalized cells. All together, our and these results confirm the ability of exogenous malignant DNA to horizontally drive transformation and tumorigenesis regardless of the model used.

The “endocrine” capacity of tumors influencing tumor progression has already being reported. McAllister et al., showed that “instigating” tumors, even when relatively small (<0.08% of total body mass), facilitate the outgrowth of already-established, otherwise-indolent tumor cells located at distant sites via the humoral factor osteopontin [43]. Our study provides evidence that tumor progression also occurs via horizontal transfer of oncogenic DNA. This is supported by the fact that human DNA (mutated *K-Ras*, *RAB* and *Alu Yd6* sequences) were found only in the tumor of the rat injected with SW480 cells and by the lower frequency of tumor formation in the rats that were treated with DNAse I/proteases which strongly suggests that this treatment decreased the levels of SW480-derived DNA in the circulation of rat that in turn reduced or avoided “passive” transfection of such DNA into rat colon epithelial cells. Ongoing research in our laboratory shows that DNAse I/protease treatment reduces the levels of circulating DNA in rats blood.

Limitations of our study are the small numbers of rats treated with DNAse I/proteases that did not allow to show a statistically significant reduction in the number of tumors as compared to rats receiving both DMH and SW480 injection; the fact that we only microdissected a single tumor from each group of rats and that we did not evaluate that these human genes (mutated *K-Ras* and *RAB*) transferred to rat colon tumors were actually expressed. It cannot be ruled out that humoral factors other than DNA could also have contributed to tumor progression as well as the potential unspecific effect of the SW480 injection into rats even that they were immunocompetent.

The results of this work need to be expanded by further research, nevertheless, we believe that the realization that circulating extracellular DNA is responsible for cancer progression may derive to the application of an antitumor therapy aimed to deplete this “oncogenic DNA”. In fact, the antitumor and antimetastatic effects of DNAse I and proteases have already being suggested [44]–[48]. The exploration of the horizontal tumor progression mediated by either DNA and/or other biologically active circulating molecules is clearly needed to determine whether its manipulation could have value in cancer therapy.

## Supporting Information

Figure S1 **Heat map representing the DNA copy number along all chromosomes.** Blue represents regions with deletions and red regions with amplifications. A nearly identical pattern of DNA copy number changes between extracellular (SpDNA SW480) and intracellular (DNA SW480) DNA, compared to a normal reference can be observed.
(PDF)

Figure S2 **Lack of transformation and DNA transfer in primary cells.** **A.** Primary human-foreskin fibroblasts (BB1) exposed for 45 days to the SW480 supernatant failed to transform and to form tumors in nude mice. Negative control was the wild-type BB1 cell line and positive control, NIH3T3 cultured with the SW480 supernatant (NIH3T3+Sp). **B.** PCR and RT-PCR of viral oncogenes of HPV-18 in several organs of Wistar rats treated every other day with intravenous injections of apoptotic bodies from HeLa cells. Transfer and expression of viral oncogenes were demonstrated to occur in liver.
(PDF)

Figure S3 **Experimental design to demonstrate horizontal tumor progression in Wistar rats.** Rats were treated with the colon carcinogen 1,2-Dimethylhydrazine (DMH) and subcutaneously (s.c.) injected with human SW480 colon cancer cells.
(PDF)

Figure S4 **Micro PET-CT using ^18^F-FDG tumor uptake in rats.** A rat receiving only DMH, **A** shows an irregular mass in the abdominal area with a SUV of 2.2 (at second evaluation). The rat receiving DMH and SW480 cells had abdominal areas of masses with a SUV of 4.1 (at second evaluation), indicating the presence of tumor (**B**).
(PDF)

Figure S5 **Histological sections of the site of inoculation of SW480 cells.** Viable tumor cells are observed at 24 h (**A**); at 72 h these are decreasing (**B**), extensive apoptosis and central necrosis are observed at 7 days (**C**). At 14 days, no viable cells were found (**D**).
(PDF)

Figure S6 **Proposed model of tumor progression mediated by horizontal DNA transfer.** The scheme at left summarizes the lateral tumor progression in rats. The SW480 xenograft sheds DNA into circulation which transforms DMH initiated colon cells to form tumors. The figure at the right is the proposed model where a primary tumor regardless of its location and type sheds “oncogenic” DNA to the circulation which “passively” transfects initiated stem cells from any site giving rise to metastases. According to this model some “metastases” can be in fact, secondary tumors.
(PDF)
