# Supplementary figures and images for: Cancer Progression Mediated by Horizontal Gene Transfer in an In Vivo Model
Source: PLoS One. 2012 Dec 28;7(12):e52754. doi: 10.1371/journal.pone.0052754 (PMC3532306; doi:10.1371/journal.pone.0052754)

SUPPL. FIGURE 1

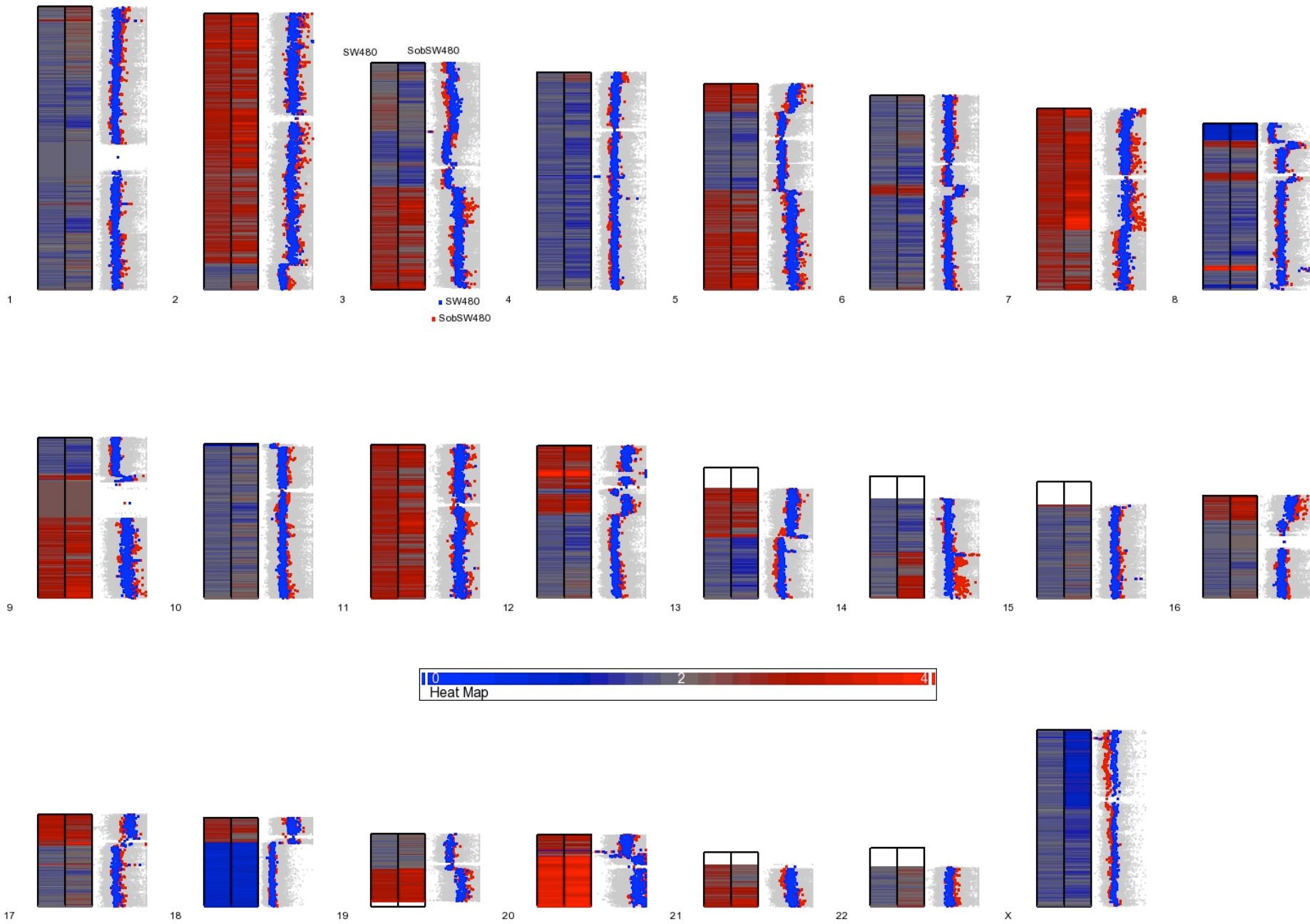

Supplement: Figure S1 — Heat map representing the DNA copy number along all chromosomes. Blue represents regions with deletions and red regions with amplifications. A nearly identical pattern of DNA copy number changes between extracellular (SpDNA SW480) and intracellular (DNA SW480) DNA, compared to a normal reference can be observed. (PDF) [file pone.0052754.s001.pdf]

SUPPL. FIGURE 2

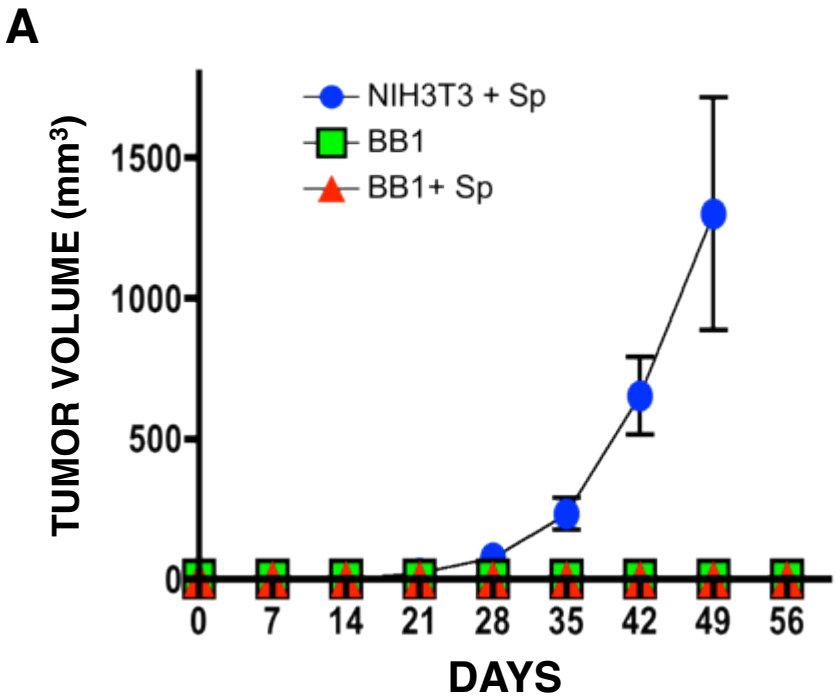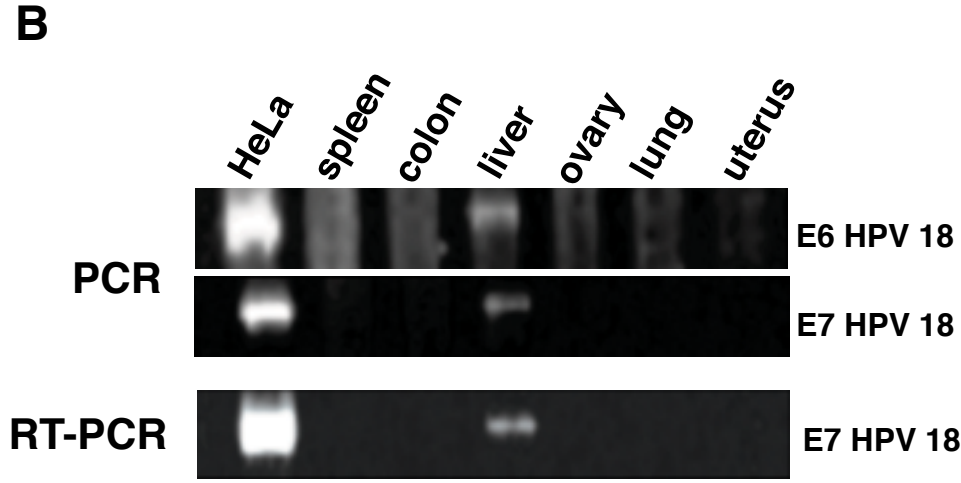

Supplement: Figure S2 — Lack of transformation and DNA transfer in primary cells. A. Primary human-foreskin fibroblasts (BB1) exposed for 45 days to the SW480 supernatant failed to transform and to form tumors in nude mice. Negative control was the wild-type BB1 cell line and positive control, NIH3T3 cultured with the SW480 supernatant (NIH3T3+Sp). B. PCR and RT-PCR of viral oncogenes of HPV-18 in several organs of Wistar rats treated every other day with intravenous injections of apoptotic bodies from HeLa cells. Transfer and expression of viral oncogenes were demonstrated to occur in liver. (PDF) [file pone.0052754.s002.pdf]

## SUPPL. FIGURE 3

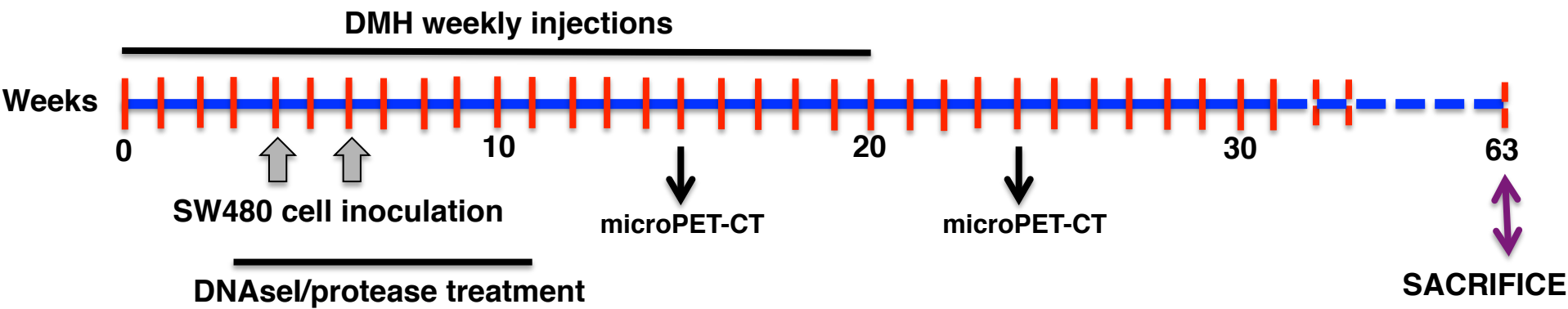

Supplement: Figure S3 — Experimental design to demonstrate horizontal tumor progression in Wistar rats. Rats were treated with the colon carcinogen 1,2-Dimethylhydrazine (DMH) and subcutaneously (s.c.) injected with human SW480 colon cancer cells. (PDF) [file pone.0052754.s003.pdf]

**SUPPL. FIGURE 4**

**A**

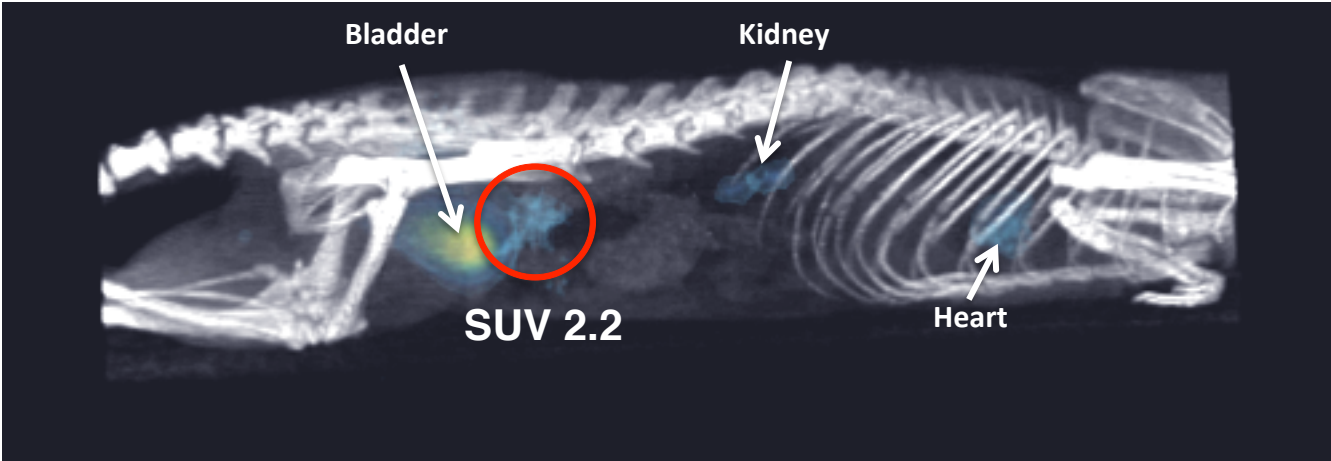

**B**

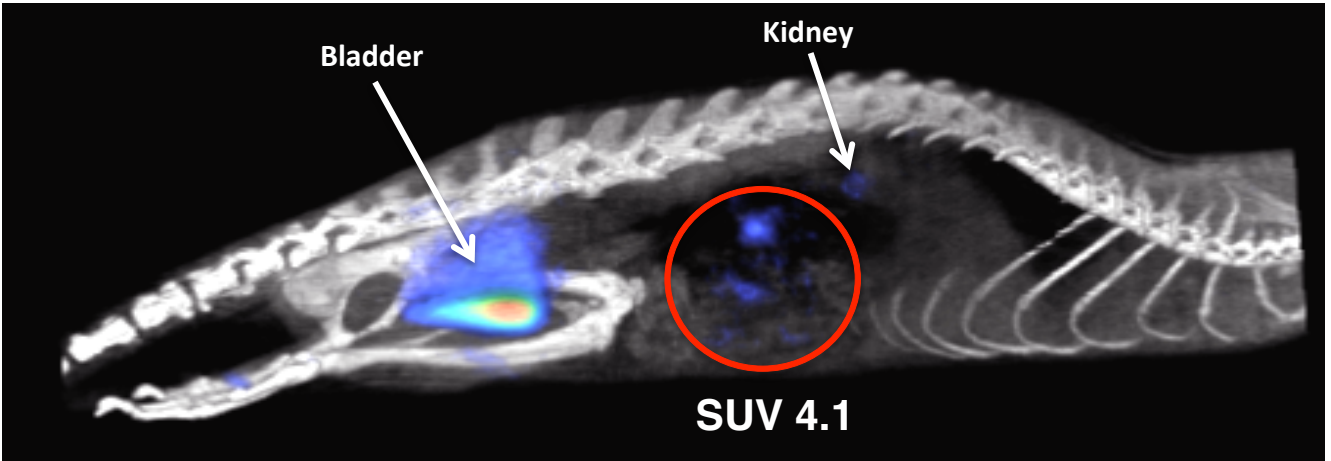

Supplement: Figure S4 — Micro PET-CT using 18F-FDG tumor uptake in rats. A rat receiving only DMH, A shows an irregular mass in the abdominal area with a SUV of 2.2 (at second evaluation). The rat receiving DMH and SW480 cells had abdominal areas of masses with a SUV of 4.1 (at second evaluation), indicating the presence of tumor (B). (PDF) [file pone.0052754.s004.pdf]

**SUPPL. FIGURE 5**

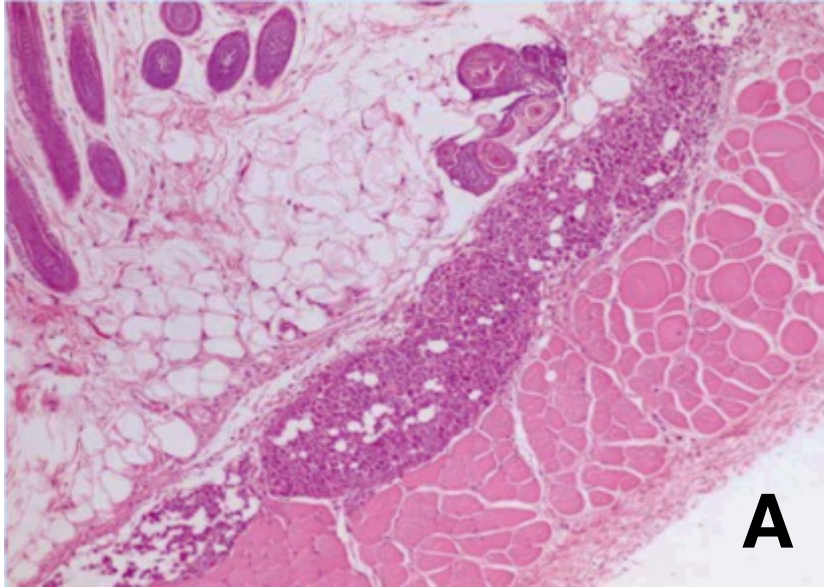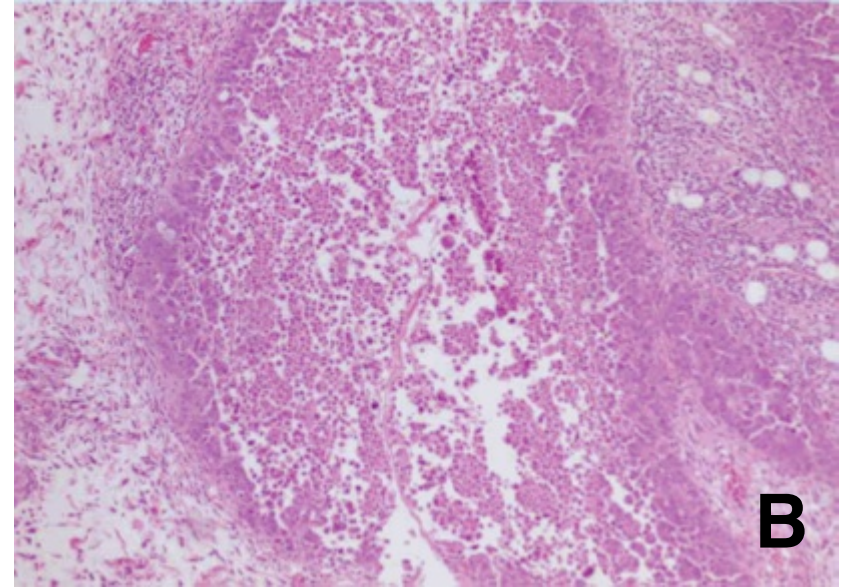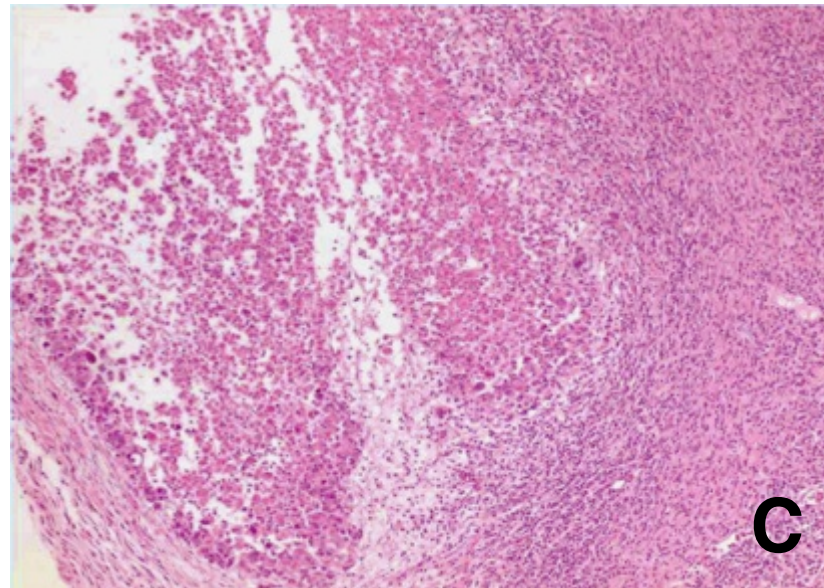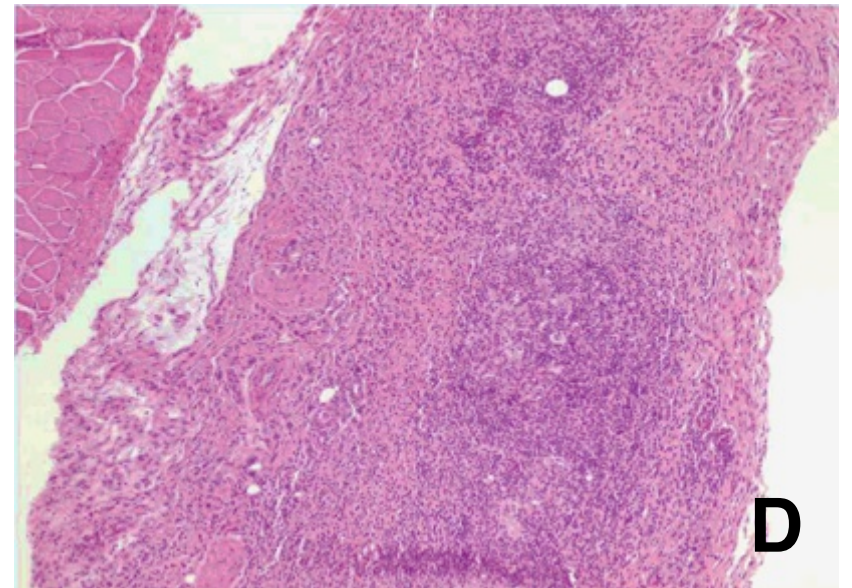

Supplement: Figure S5 — Histological sections of the site of inoculation of SW480 cells. Viable tumor cells are observed at 24 h (A); at 72 h these are decreasing (B), extensive apoptosis and central necrosis are observed at 7 days (C). At 14 days, no viable cells were found (D). (PDF) [file pone.0052754.s005.pdf]
