# Supplementary material for: Cancer Progression Mediated by Horizontal Gene Transfer in an In Vivo Model
Source: PLoS One. 2012 Dec 28;7(12):e52754. doi: 10.1371/journal.pone.0052754 (PMC3532306; doi:10.1371/journal.pone.0052754)

SUPPL. FIGURE 6

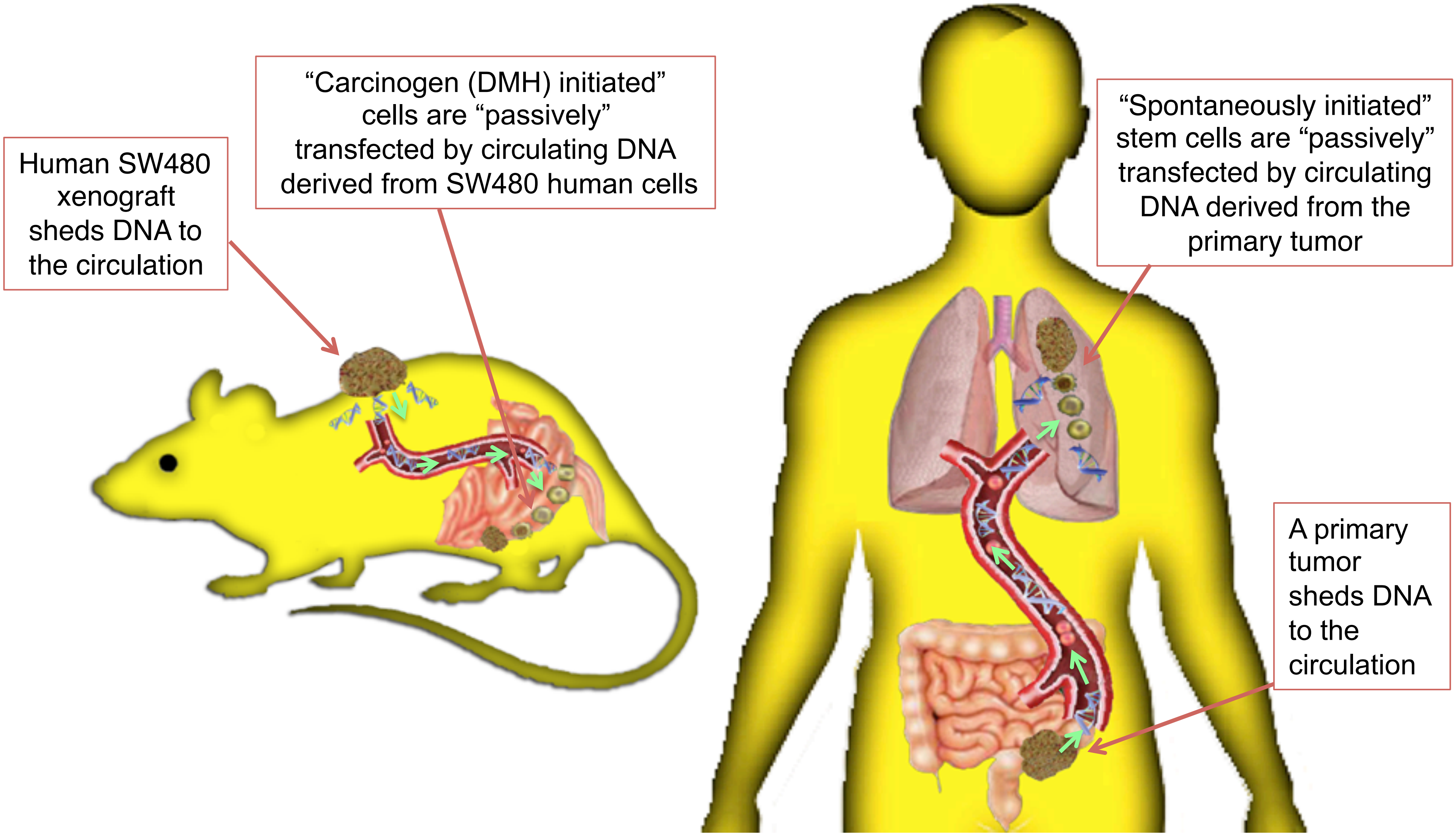

Supplement: Figure S6 — Proposed model of tumor progression mediated by horizontal DNA transfer. The scheme at left summarizes the lateral tumor progression in rats. The SW480 xenograft sheds DNA into circulation which transforms DMH initiated colon cells to form tumors. The figure at the right is the proposed model where a primary tumor regardless of its location and type sheds “oncogenic” DNA to the circulation which “passively” transfects initiated stem cells from any site giving rise to metastases. According to this model some “metastases” can be in fact, secondary tumors. (PDF) [file pone.0052754.s006.pdf]
